# Loss and gain of N-linked glycosylation sequons due to single-nucleotide variation in cancer

**DOI:** 10.1038/s41598-018-22345-2

**Published:** 2018-03-12

**Authors:** Yu Fan, Yu Hu, Cheng Yan, Radoslav Goldman, Yang Pan, Raja Mazumder, Hayley M. Dingerdissen

**Affiliations:** 1 The Department of Biochemistry & Molecular Medicine, The George Washington University Medical Center, Washington, DC 20037 United States of America; 2 Department of Oncology, Georgetown University, Washington, DC 20057 United States of America; 3 McCormick Genomic and Proteomic Center, The George Washington University, Washington, DC 20037 United States of America

## Abstract

Despite availability of sequence site-specific information resulting from years of sequencing and sequence feature curation, there have been few efforts to integrate and annotate this information. In this study, we update the number of human N-linked glycosylation sequons (NLGs), and we investigate cancer-relatedness of glycosylation-impacting somatic nonsynonymous single-nucleotide variation (nsSNV) by mapping human NLGs to cancer variation data and reporting the expected loss or gain of glycosylation sequon. We find 75.8% of all human proteins have at least one NLG for a total of 59,341 unique NLGs (includes predicted and experimentally validated). Only 27.4% of all NLGs are experimentally validated sites on 4,412 glycoproteins. With respect to cancer, 8,895 somatic-only nsSNVs abolish NLGs in 5,204 proteins and 12,939 somatic-only nsSNVs create NLGs in 7,356 proteins in cancer samples. nsSNVs causing loss of 24 NLGs on 23 glycoproteins and nsSNVs creating 41 NLGs on 40 glycoproteins are identified in three or more cancers. Of all identified cancer somatic variants causing potential loss or gain of glycosylation, only 36 have previously known disease associations. Although this work is computational, it builds on existing genomics and glycobiology research to promote identification and rank potential cancer nsSNV biomarkers for experimental validation.

## Introduction

Protein glycosylation is the enzymatic process by which a carbohydrate is covalently attached to a target protein, and is a form of co-translational or post-translational modification (PTM)^1^. It is the most complex protein modification due to the diversity of glycans and possible branching configurations^2^, and it impacts protein characteristics, including folding, stability, cell motility, and cell-cell adhesion^1,3,4^, that could be implicated in disease. Glycosylation is an enzyme-directed, site-specific process: a complex network of enzymatic pathways is responsible for the broad variety of glycan structures, and the site-specific structural diversity of glycosylated proteins determines functionality^1,4^.

Glycoproteins belong primarily to two groups: N-linked and O-linked^1,5^. N-glycans are covalently linked to the carboxamide (-CONH2) group of an asparagine residue in the first position of the specific N-linked glycosylation sequon (NLG), NXS/T (X! = P), where N is asparagine, S/T denotes either serine or threonine, and X is any amino acid except proline^6^. Occasionally, glycosylation may occur when a cysteine (C) is in the third position of the sequon instead of an S or T^7,8^. Not all NLGs are glycosylated due to the limited accessibility and intricate regulation of glycosylation-related enzymes^9^, and other sequence motifs for the attachment of N-glycans have been described but are utilized with much lower frequency^10^.

While the clinical relevance of glycosylation was reported several years ago^11^, the last two decades have seen an increase of human glycosylation profiling studies due to technological advances including high-throughput sequencing (HTS)^12–15^ and glycomics approaches^16–19^. A number of sequence and feature annotation databases now exist^20–25^, including the Universal Protein Knowledgebase (UniProtKB)^26^, which provides a consistent and richly annotated repository of protein function, interaction, disease relatedness, and other characteristics^26^. The Swiss-Prot section of UniProtKB provides additional information concerning biological pathways, PTMs, and disease variants^27^, collected by multiple approaches^28,29^ and subject to manual review by experts.

Using data available through UniProtKB/Swiss-Prot, Apweiler *et al*. published a comprehensive study about the distribution of NLGs across all organisms in 1999^30^, reporting that almost two thirds of proteins contained the NX(S/T) (X! = P) sequon, and predicting that most sequon-containing proteins would eventually be confirmed as true glycoproteins. According to release statistics, the amount of protein entries and associated annotations in UniProtKB/Swiss-Prot has since drastically changed: there are currently more than 550,000 UniProtKB/Swiss-Prot entries representing more than 10,000 species, with humans being the most represented species with respect to number of protein entries. Despite this growth, there has not yet been a formal update of the NLG distribution in humans. Thus, our first research objective for this study was to use current data to provide new statistics regarding the distribution and occupancy rate of NLGs among human proteins. Note that although glycosylation has been observed at NXC sites, we limit the scope of this study to the traditional NXS/T sequon at this time due to the relative rarity of verified glycosylation at atypical sequons.

While loss and gain of glycosylation can be a factor during evolution and is not necessarily damaging^31^, specific changes in human glycosylation have been associated with pathology or physiology of processes like embryonic development or carcinogenesis and can be explored efficiently as biomarkers^1,32–34^. Notable changes observed in glycan profiles are a consequence of environmental influences and physiologic responses and therefore could have a significant diagnostic potential^3^. For example, the presence of certain glycoconjugate species has been shown to interact with and potentially regulate cancer cell processes, tumor malignancy, and tumor microenvironment^35–38^.

Specifically, variations impacting NLGs could directly cause disease by altering glycosylation; this hypothesis is consistent with the observations that the majority of human inherited disease missense mutations affect protein stability^39^, and that N-linked glycosylation decreases protein dynamics and likely increases stability^40^. In fact, there are a number of cases demonstrating the role of loss and gain of glycosylation in disease. For example, the loss of an NLG in the prion protein (PRNP) due to a T183A substitution leads to spongiform encephalopathy^41,42^, and sequon loss by a T315A in Vitamin-K dependent protein C is associated with venous thromboembolism^43^. Conversely, a gain of glycosylation results from the well-characterized D356N germline variation in the sex hormone-binding globulin protein (SHBG), which generates an additional N-linked site that has been observed with an attached carbohydrate chain. This variation and gain of SHBG glycosylation corresponds to a decreased SHBG clearance rate, an increased half-life, and increased circulating levels of the protein, both in humans and animals^44–46^. Additionally, sequon gain via A349S in Integrin alpha-3 and its subsequent hyperglycosylation results in multiorgan failure^47^. Although variations involving gain of glycosylation sequons (GOGs) have been considered rare, up to 1.4% of known disease-causing missense mutations involving human genetic diseases can lead to GOGs; in some cases, attachment of novel glycans at these sites is sufficient to account for the detrimental effect of the corresponding variation^48,49^.

The above-referenced technological advances have paved the way for studies concerning the functional importance of N-linked glycosylation in disease, including cancer^35,50^. There is now evidence that β1,6GlcNAc-branching of N-glycans directly contributes to cancer progression, and both GlcNAc-branched N-glycans and terminal Lewis antigen sequences have been observed to increase in some cancers and are related with poor prognosis^51^. In breast cancer, the frequency of the aforementioned SHBG D356N variant was significantly higher in estrogen-dependent cases and suggested a close association between the additionally glycosylated variant and the estrogen-dependence of breast cancer^52^. Interestingly, this variant is distributed with different frequencies among different ethnic groups^53^. Thus, our second major research objective of this study was to provide a pan-cancer view of the impact of nucleotide variation on the human N-linked glycosylation profile.

Restated, we conducted a comprehensive analysis of nsSNVs that may lead to either loss or gain of NLGs and compared functional impacts of germline SNPs and somatic nsSNVs, especially with respect to cancer involvement. While independent genomics databases provide variation data from cancer samples and literature mining tools can extract disease and variation information, this work is the first comprehensive effort toward the integration of N-linked glycosylation data with nsSNVs from multiple cancer genomics databases with value-added information and high-value curations from additional resources (such as dbSNP, ClinVar, UniProtKB/Swiss-Prot, and publications). Integrated data were then mapped to Disease Ontology (DO)^54^ terms to facilitate a comparative functional impact analysis^55^ of nucleotide variation. Synthesis of this information allowed for ranking proteins for which variation may affect N-glycosylation in cancer. We suggest high-confidence proteins and/or variants represent ideal candidates for downstream validation with respect to both glycosylation status and cancer involvement. Note that while we do prioritize these variants based on relevance of functional annotations and presence in multiple cancers for the purposes of targeted future study, we do not distinguish between the roles of glycosylation-impacting variants across multiple cancers or within a distinct cancer type.

## Results

### Comprehensive collection of real and predicted NLGs

#### Overview of human NLGs

Table 1 summarizes the findings from each of the three methods of NLG identification: high-confidence NLGs (those reported in databases with validated evidence or manual assertion), predicted NLGs by NetNGlyc (http://www.cbs.dtu.dk/services/NetNGlyc/), and string search of all NX(S/T) (X! = P) sequons. Proteins predicted to contain signal peptides or annotated with the UniProt keyword(s) “Secreted” or “Membrane” were used for analysis of disease-relatedness to focus on those entries with the greatest potential of being biologically viable biomarkers. Of the 59,341 identified non-redundant NLGs from 15,318 proteins (See Supplemental Table S1), 7,017 of these proteins either have signal peptides or are annotated with one or both keyword(s) “Secreted” or “Membrane,” prioritizing this subset for consideration as biologically viable markers. Note that of the 15,318 total proteins identified to contain NLGs and the subset of 7,017 likely viable N-linked glycosylation sites identified by functional annotations, 15,314 and 7,014, respectively, were retrievable by the string search method alone. Additional information about these NLG-containing sequences, including sequence length, existence and positions of signal peptides, “Cellular component” keywords, and more, can be viewed in Supplemental Table S1.

**Table 1:** Numbers of sequons and proteins identified from three methods 3,452 out of 20,199 proteins in the human proteome have signal peptides; 14,921 proteins in the human proteome have at least one “Cellular component” keyword (“Secreted,” “Membrane,” “Cytoplasm,” or “Nucleus”); the same protein can belong to multiple “Cellular component” categories if it is observed in more than one cellular location. 8,772 proteins have either signal peptides or are annotated with keyword(s) “Secreted” or “Membrane.” String search results include almost all the NLGs from the other two methods except for 121 atypical cases which do not follow consensus NX(S/T) (X! = P) according to high-confidence criterion, 61 reported from UniProt FT lines. There are 59,341 non-redundant NLGs from 15,318 proteins in total from these three methods. 7,017 of them either have signal peptides or are annotated with keyword(s) “Secreted” or “Membrane”.

	High-confidence^a^ NLGs from Databases	Predicted NLGs by NetNGlyc	String search of NX(S/T) (X! = P) sequons
Sequons	16,253	43,139	59,220
Proteins	4,412	14,114	15,314
Proteins either have signal peptides or annotated with keyword(s) “Secreted” or “Membrane”	4,373	6,597	7,014

^a^Annotated NLGs from UniProtKB/Swiss-Prot, HPRD 9.0, dbPTM 3.0, neXtProt and NCBI-CDD were treated as high-confidence results.

#### Frequency of NLGs and experimentally validated sites in human proteins

**Figure 1:**
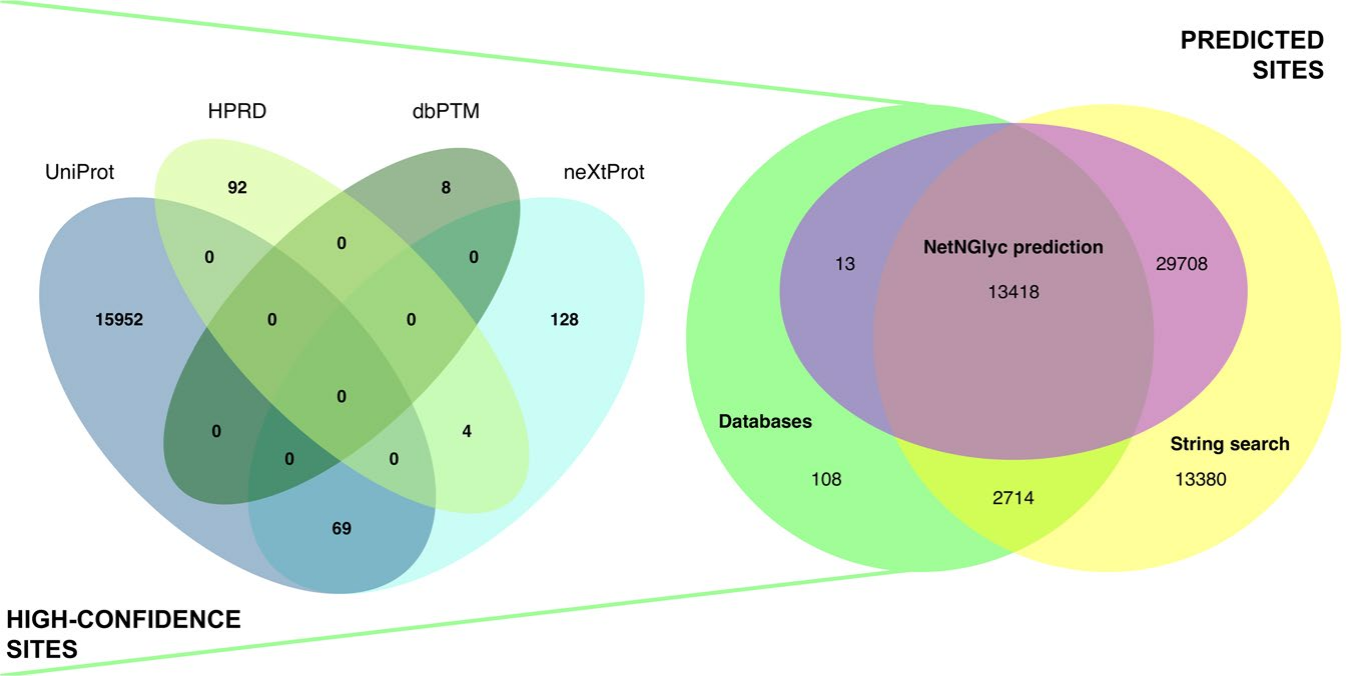
Contribution of data sources to NLG identification. This diagram shows the distinct and overlapping contribution of different resources and methods of identification of NLGs throughout the human proteome. The contributions of specific databases (those data entries composing the high-confidence subset) are detailed in the Venn diagram on the left.

Within the UniProtKB/Swiss-Prot dataset, there are 15,318 of 20,199 human proteins, or 75.8%, of all human proteins with at least one NLG. Of the 59,341 NLGs on these human proteins, for an average of 3.9 per protein, 16,253 of 21,956, or 74.03% of total NLGs from 4,412 glycoproteins are well-characterized N-linked glycosylation sites. In this context, the term glycoprotein is applied based on the observation of at least one occupied NLG. Among the high-confidence sites, 2,511 NLGs are experimentally verified with evidence from publications and 13,500 are manually asserted by UniProtKB/Swiss-Prot curators. 232 additional NLGs were retrieved from other databases and are included in the table of potential NLGs (see Materials and Methods section). Figure 1 shows the relative contribution of sources to the identification of NLGs. Furthermore, 61 NLGs belonging to these high-confidence N-linked glycosylation sites are atypical in that the corresponding UniProtKB/Swiss-Prot FT line annotations do not follow the consensus NX(S/T) (X! = P) configuration. Therefore, although the current rate of experimentally validated human NLGs is 27.39%, the rate of experimentally validated NLGs in the subset of known glycoproteins is 74.03%, aligning with Apweiler’s previous hypothesis that three quarters of glycoproteins should be N-linked.

#### Distribution and spacing of NLGs and experimentally validated N-linked glycosylation sites

**Figure 2:**
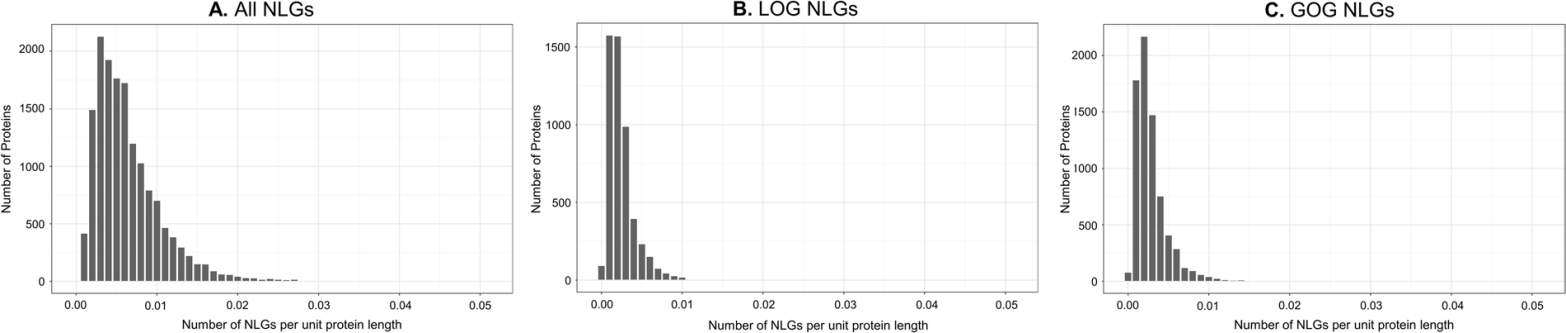
Density of sequons per protein. Density here is calculated as the total number of positions identified to start an NXS/T motif in a protein divided by the corresponding length of the protein. The majority of proteins are annotated with smaller numbers of NLGs, and therefore the average density is less than 1 NLG per protein, when normalized by unit length. The distribution of NLGs per protein is plotted as the count of proteins with a given density of NLGs for (**A**) all NLGs in the human proteome, (**B**) LOG-causing NLGs in the somatic subset, and **C**) GOG-causing NLGs in the somatic subset.

The distribution of NLGs per protein is shown in Fig. 2 (for more information, see Supplemental Table S2). Most proteins have a small number of NLGs, but some proteins have large numbers of NLGs. The most extreme example of this case is Mucin-16 (MUC16, UniProtKB accession: Q8WXI7), which has 265 NLGs. As the largest cell-associated mucin, the length of MUC16 at 22,152 residues^56^ increases the likelihood for multiple NLGs in the sequence. Mucins are a major component of mucus with a normal protective physiological role, but it has been shown that glycan attachment to MUC16 is altered in response to oxidative stress in pancreatic cancer^57^ and is a major carrier of altered sialylation characteristic of malignant conditions in serous ovarian tumors^58^. When normalized by length, MUC16 still shows a greater than average number of NLGs per unit length (Table 2), but Sialomucin core protein 24 (CD164, Q04900) has the greatest density of NLGs at approximately one per every 22 residues, compared to the average of approximately one per every 118 residues. CD164 belongs to a class of heavily glycosylated proteins, the sialomucins, involved in regulation of cellular adhesion, proliferation, differentiation, and migration of hematopoietic stem cells^59^. Although its expression, not glycosylation, has been implicated in various cancers^60,61^, absence of proper terminal N-glycan attachment on CD164 protein has been observed to prevent functional interactions, disrupting post-endocytic sorting of the protein^59^. Table 2 demonstrates similar trends in the spacing of both total NLGs and verified N-linked glycosylation sites when averaged for all proteins.

**Table 2:** Spacing of sequons and real N-glycosylation sites. All values are reported as numbers of amino acids.

	Sequons	Real N-glycosylation sites
Minimum	1	1
Maximum	4,277	2,970
Mean	117.98	93.84
Median	71	52
S.D.	145.02	134.71

### Summary of collected variation data

A total of 1,272,878 somatic nsSNVs collected across cancer genomics databases are associated with 59 cancer types which are mapped to the Cancer Disease Ontology (CDO)^62^. There are also 3,680,786 germline SNPs from dbSNP.

### Identification of LOG and GOG resulting from variation

Mapping of nsSNVs to NLGs led to identification of variations that may lead to loss of glycosylation (LOG) and gain of glycosylation (GOG). In some cases, different variations can lead to abolition or creation of the same NLG, and proteins may contain multiple NLGs. Thus, we expect the number of variations to be greater than or equal to the number of affected sequons, which is expected to be greater than or equal to the number of affected proteins. Our observations follow this trend, as can be seen in Table 3 (additional information about LOG and GOG variations and corresponding sequons can be found in Supplemental Tables S3–S8).

**Table 3:** Numbers of variations, affected sequons, and affected proteins from germline and cancer somatic LOG and GOG variation.

	Somatic			Somatic-only^a^			Germline		
	**nsSNVs**	**Sequons** ^b^	**Proteins**	**nsSNVs**	**Sequons** ^**b**^	**Proteins**	**nsSNVs**	**Sequons** ^b^	**Proteins**
LOG due to variations within the NX(S/T) (X! = P) sequon	10,807	9,665	5,893	8,895	8,061	5,204	37,498	26,909	10,586
GOG due to variations within the NX(S/T) (X! = P) sequon^c^	15,675	15,503	8,257	12,939	12,815	7,356	39,455	38,711	12,667
GOG predicted by NetNGlyc due to variations^d^	11,018	10,918	6,808	9.835	9.294	5,365	27,704	27,226	11,307

^a^Variations from cancer genomics databases (somatic origin), but not in dbSNP (germline origin)

^b^Sequons for GOG sets are reported by unique positions- Note that the number of unique motifs per position identified is equal to the total number of nsSNVs for that set.

^c^GOG predicted by string search alone

^d^Overlap between string search and NetNGlyc is 100% of NetNGlyc results

**Figure 3:**
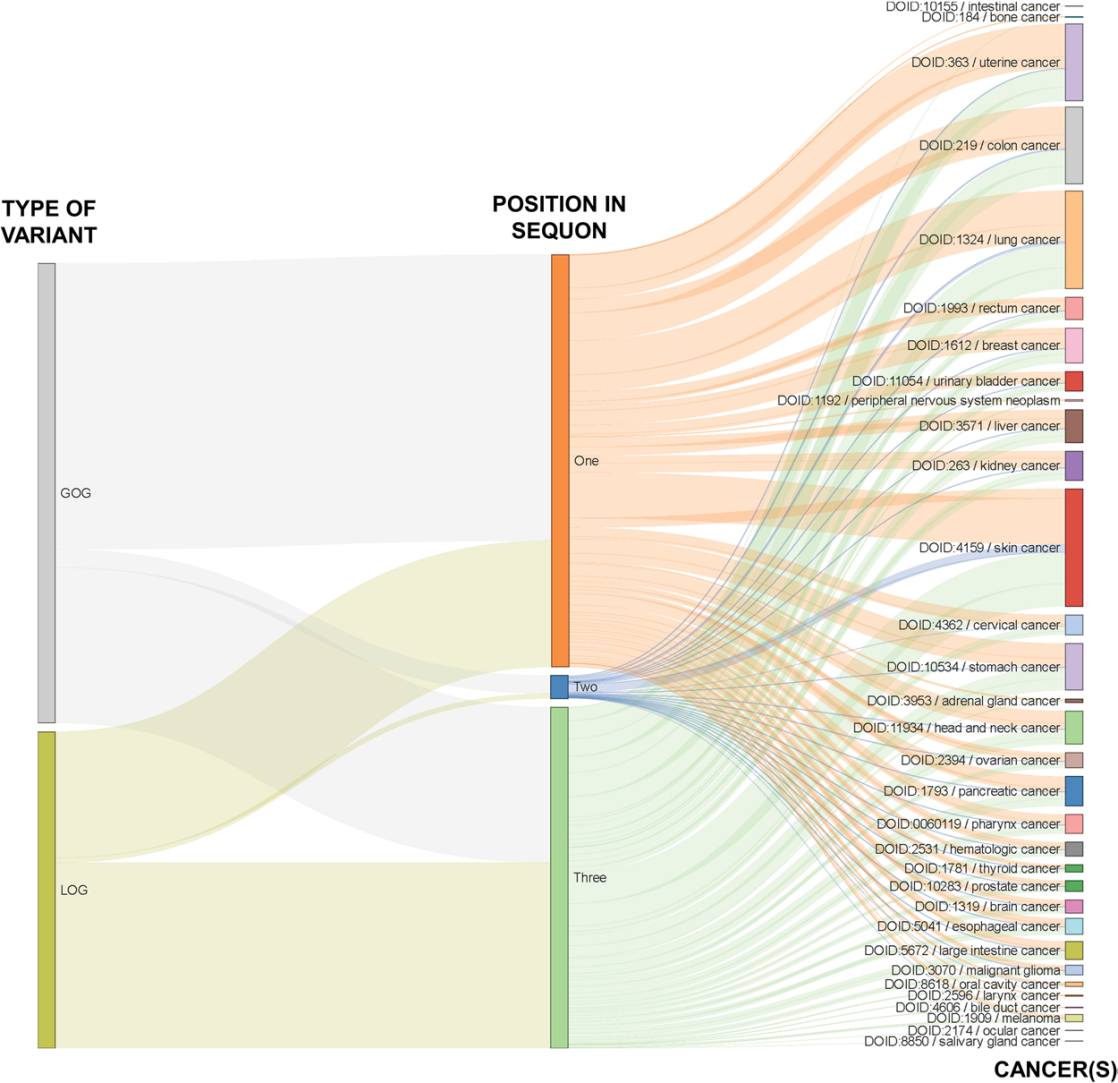
Position of affected residue in sequon in LOG and GOG cancer subset. LOG and GOG variants are linked to the position in the sequence affected by the underlying nsSNV, where one corresponds to N, two corresponds to X, and three corresponds to S/T in the NXS/T consensus sequon. Variants are also linked to the specific cancers in which they have been identified.

In this study, we report 16,253 high-confidence (experimentally validated or manually asserted) sites with 2,898 and 7,508 sequons abolished by somatic and germline variations, respectively. Although experimental validation of GOG is outside the scope of this study, we report 15,504 and 38,711 potentially induced sequons by somatic and germline variations, respectively. Furthermore, in this update we report that 3,930 of 4,412 high-confidence glycoproteins lose or gain NLGs due to germline or cancer somatic nsSNV compared to 1,091 human proteins previously reported to undergo LOG or GOG due to polymorphisms^63^. For all datasets, the second position in the sequon is the least subject to variations that would induce or abolish a sequon, as is expected due to the flexibility of residues at this position (can be any residue except P). Conversely, the first position requires an N, and is therefore expected to be the most subject to NLG-altering mutations. The third position, which can be occupied by either an S or a T, is neither as limited as the first position nor as flexible as the second, so we expect the frequency of sequon-altering variations to be between the corresponding frequencies for the first and second positions. When we average across all datasets, these trends do hold true; however, when we look at just the subset of somatic LOG mutations, we actually find that the third position is subject to a greater frequency of mutations than the first position (Fig. 3).

### Proteome-wide pan-cancer analysis

#### Significant variations

**Figure 4:**
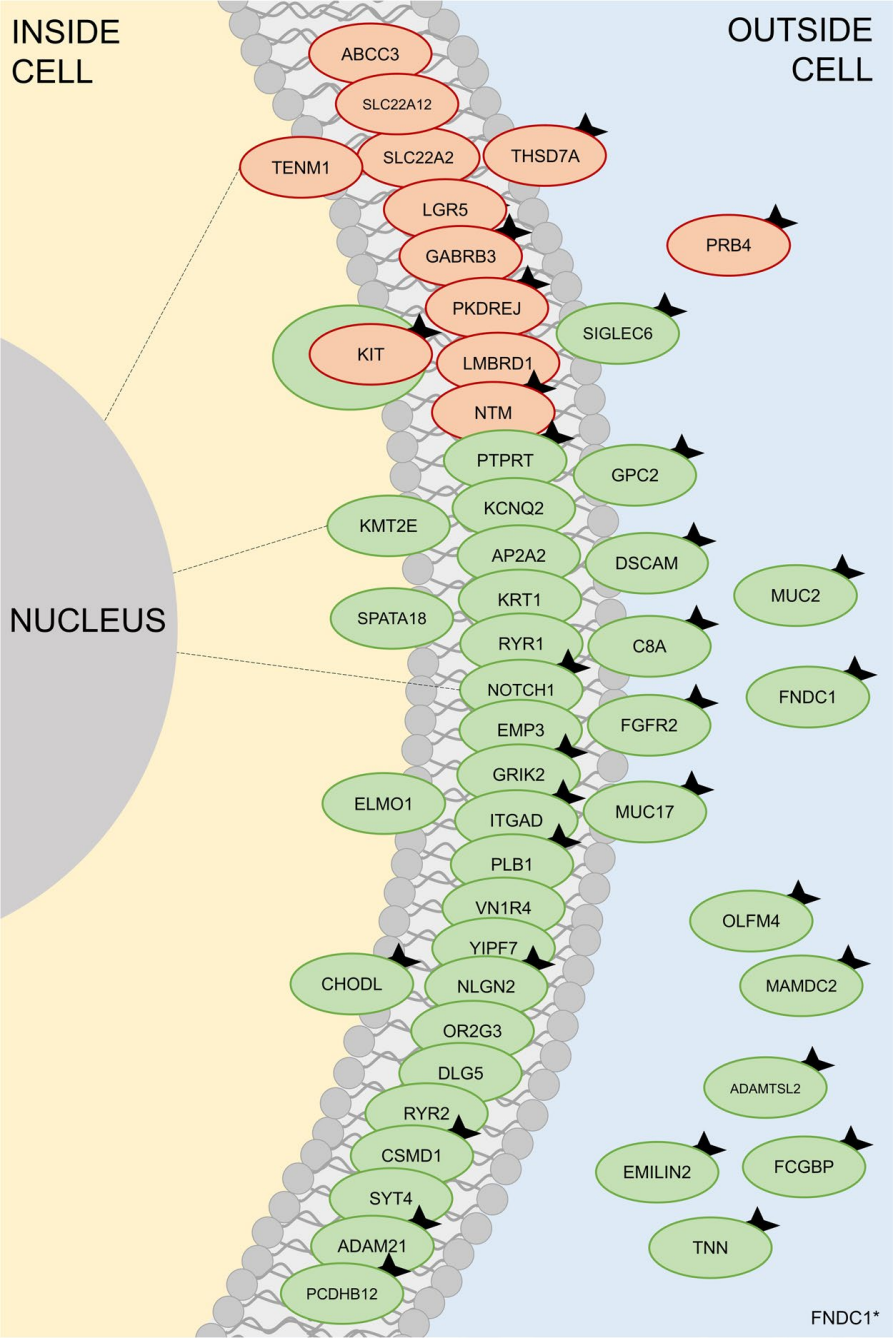
Schematic of biologically relevant LOG and GOG variations in at least three cancers. Red circles are proteins in the LOG dataset, green circles are proteins in the GOG dataset. Protein completely within the lipid bilayer are tagged with cellular localization term “Membrane” while proteins spanning both the membrane and the adjacent cytoplasm or extracellular environment are tagged both with cellular localization term “Membrane” and “Cytoplasm” or “Secreted,” respectively. Note that position within membrane is not indicative of status as integral or peripheral proteins. Dotted lines represent proteins that also have cellular localization term “Nucleus.” Black stars on proteins signify the presence of a signal peptide on that protein. Also, note that Mast stem cell growth factor receptor Kit, (KIT, P10721) is the only protein to appear with mutations that could cause loss or gain of glycosylation in more than three cancers each.

Somatic-only LOG/GOG-causing variations are associated with 38 cancer types. For proteins either having signal peptides or annotated with keyword(s) “Secreted” or “Membrane,” we found 24 LOG-causing somatic-only variations in 24 sequons of 23 proteins and 41 GOG-causing somatic-only variations in 41 sequons of 40 proteins present in at least three cancer types (See Supplemental Tables S7–S10). If we only include high-confidence NLGs reported in databases, we find 13 LOG-causing somatic-only variations in 13 sequons of 12 proteins. Figure 4 shows a schematic of the localization of these proteins.

Interestingly, considering only the subset of high-confidence NLGs, there are 13 somatic-only LOG variations associated with three or more cancer types each (Table 4), two of which are associated with four or more cancer types each. Under these criteria, the most significant variations are Mast/stem cell growth factor receptor Kit (KIT, P10721) N486D and Thrombospondin type-1 domain-containing protein 7A (THSD7A, Q9UPZ6) T236M.

**Table 4:** High-confidence LOG variations related with three or more cancer types.

UniProt AC	Gene Name	Protein Name	Position	Reference	Variation	Cancer Types
O15244	SLC22A2	Solute carrier family 22 member 2	74	T	M	DOID:1793/pancreatic cancer;DOID:1319/brain cancer;DOID:219/colon cancer
O15438	ABCC3	Canalicular multispecific organic anion transporter 2	1009	S	F	DOID:4159/skin cancer;DOID:10534/stomach cancer;DOID:3571/liver cancer
O75473	LGR5	Leucine-rich repeat-containing G-protein coupled receptor 5	77	N	S	DOID:219/colon cancer;DOID:1324/lung cancer;DOID:5672/large intestine cancer
P10163	PRB4	Basic salivary proline-rich protein 4	110	S	P	DOID:1319/brain cancer;DOID:3070/malignant glioma;DOID:11934/head and neck cancer
P10721	KIT	Mast/stem cell growth factor receptor Kit	486	N	D	DOID:184/bone cancer;DOID:1993/rectum cancer;DOID:219/colon cancer;DOID:5672/large intestine cancer
P28472	GABRB3	Gamma-aminobutyric acid receptor subunit beta-3	107	T	M	DOID:0060119/pharynx cancer;DOID:10534/stomach cancer;DOID:11934/head and neck cancer
Q96S37	SLC22A12	Solute carrier family 22 member 12	104	T	M	DOID:1319/brain cancer;DOID:10534/stomach cancer;DOID:219/colon cancer
Q9NTG1	PKDREJ	Polycystic kidney disease and receptor for egg jelly-related protein	297	S	L	DOID:4159/skin cancer;DOID:0060119/pharynx cancer;DOID:11934/head and neck cancer
Q9NTG1	PKDREJ	Polycystic kidney disease and receptor for egg jelly-related protein	925	S	L	DOID:10534/stomach cancer;DOID:219/colon cancer;DOID:5672/large intestine cancer
Q9NUN5	LMBRD1	Probable lysosomal cobalamin transporter	349	S	C	DOID:1319/brain cancer;DOID:1324/lung cancer;DOID:3070/malignant glioma
Q9P121	NTM	Neurotrimin	46	T	M	DOID:0060119/pharynx cancer;DOID:5041/esophageal cancer;DOID:11934/head and neck cancer
Q9UKZ4	TENM1	Teneurin-1	1759	S	L	DOID:4362/cervical cancer;DOID:0060119/pharynx cancer;DOID:11934/head and neck cancer
Q9UPZ6	THSD7A	Thrombospondin type-1 domain-containing protein 7A	236	T	M	DOID:0060119/pharynx cancer;DOID:1993/rectum cancer;DOID:5041/esophageal cancer;DOID:11934/head and neck cancer

KIT is a transmembrane receptor tyrosine kinase commonly expressed in hematopoietic progenitor cells^64^. Inhibition of N-linked glycosylation of KIT has been reported to affect cellular signaling and cell-surface expression of KIT, inducing apoptosis in acute myeloid leukemia (AML)^65^, and glucose metabolism mediated by KIT in response to imatinib has been used to predict tumor sensitivity to the drug in gastrointestinal stromal tumors (GIST)^66^. Evidence suggests that stem cell factor, the activator of KIT’s autophosphorylation domain, only operates on a mature version of the protein with the appropriate N-linked glycosylation profile^67^. In our dataset, the N486D KIT variant was observed in bone cancer [DOID:184], rectum cancer [DOID:1993], colon cancer [DOID:219], and large intestine cancer [DOID:5672].

The status of THSD7A as an N-glycoprotein has been well documented, and inhibition of glycosylation associates with a lack of soluble THSD7A, the form known to promote endothelial cell migration in normal embryonic angiogenesis^68^. Interestingly, the involvement of all THSD7A LOG variations in cancer, including the T236M associated with four cancers (pharynx cancer [DOID:0060119], rectum cancer [DOID:1993], esophageal cancer [DOID:5041], and head and neck cancer [DOID:11934]), were all inferred from inclusion in ICGC, TCGA, COSMIC, and Intogen, and no literature citations were found for THSD7A variants involved in cancer, manually or through mapping to HGMD (Human Gene Mutation Database). However, THSD7A has been reported to map to the FRA7B common fragile site (CFS); CFS are known to correlate with recurrent breakpoints in some cancers^69^. While the lack of literature support for cancer involvement could indicate a lack of importance of THSD7A variation in cancer, this variant’s presence in four different cancers reported across four cancer databases (as listed above), in conjunction with its link to CFS, warrants additional examination. Thus, THSD7A represents a prime candidate for downstream validation of cancer involvement.

Because newly created NLGs are not expected to be experimentally verified glycosylation sites described in publications, we limit the scope of potential GOGs by relevant cellular compartment keyword and signal peptide annotation. Under these criteria, there are 41 somatic-only GOG-causing variants related to three or more cancers each (Table 5), six of which are related to four or more cancers each, and two related to five or more cancers each. These top two variations are Neurogenic locus notch homolog protein 1 (NOTCH1, P46531) A465T and Mucin-2 (MUC2, Q02817) T1750N.

**Table 5:** Somatic-only GOG variations related with three or more cancer types.

UniProt AC	Gene Name	Protein Name	Position	Reference	Variation	Cancer Types
O14522	PTPRT	Receptor-type tyrosine-protein phosphatase T	281	A	T	DOID:1319/brain cancer;DOID:10534/stomach cancer;DOID:3070/malignant glioma
O43526	KCNQ2	Potassium voltage-gated channel subfamily KQT member 2	785	D	N	DOID:1793/pancreatic cancer;DOID:1612/breast cancer;DOID:10283/prostate cancer
O43699	SIGLEC6	Sialic acid-binding Ig-like lectin 6	251	A	T	DOID:1319/brain cancer;DOID:219/colon cancer;DOID:3070/malignant glioma
O60469	DSCAM	Down syndrome cell adhesion molecule	213	A	T	DOID:363/uterine cancer;DOID:1612/breast cancer;DOID:1793/pancreatic cancer;DOID:5041/esophageal cancer
O94973	AP2A2	AP-2 complex subunit alpha-2	115	A	T	DOID:1612/breast cancer;DOID:219/colon cancer;DOID:5672/large intestine cancer
P04264	KRT1	Keratin, type II cytoskeletal 1	248	D	N	DOID:4159/skin cancer;DOID:2394/ovarian cancer;DOID:5672/large intestine cancer
P07357	C8A	Complement component C8 alpha chain	169	D	N	DOID:4159/skin cancer;DOID:363/uterine cancer;DOID:1909/melanoma
P10721	KIT	Mast/stem cell growth factor receptor Kit	566	N	S	DOID:1993/rectum cancer;DOID:263/kidney cancer;DOID:5672/large intestine cancer
P21802	FGFR2	Fibroblast growth factor receptor 2	659	K	N	DOID:363/uterine cancer;DOID:1612/breast cancer;DOID:1324/lung cancer
P21817	RYR1	Ryanodine receptor 1	2861	D	N	DOID:8618/oral cavity cancer;DOID:1793/pancreatic cancer;DOID:363/uterine cancer;DOID:11934/head and neck cancer
P46531	NOTCH1	Neurogenic locus notch homolog protein 1	465	A	T	DOID:219/colon cancer;DOID:3070/malignant glioma;DOID:5041/esophageal cancer;DOID:11934/head and neck cancer;DOID:0060119/pharynx cancer;DOID:1319/brain cancer
P54852	EMP3	Epithelial membrane protein 3	42	D	N	DOID:8618/oral cavity cancer;DOID:0060119/pharynx cancer;DOID:11934/head and neck cancer
Q02817	MUC2	Mucin-2	1750	T	N	DOID:1612/breast cancer;DOID:3571/liver cancer;DOID:1781/thyroid cancer;DOID:2394/ovarian cancer;DOID:10283/prostate cancer;DOID:1319/brain cancer;DOID:1993/rectum cancer
Q13002	GRIK2	Glutamate receptor ionotropic, kainate 2	528	D	N	DOID:8618/oral cavity cancer;DOID:1612/breast cancer;DOID:11054/urinary bladder cancer
Q13349	ITGAD	Integrin alpha-D	1070	D	N	DOID:363/uterine cancer;DOID:1993/rectum cancer;DOID:219/colon cancer;DOID:5672/large intestine cancer
Q4ZHG4	FNDC1	Fibronectin type III domain-containing protein 1	253	K	N	DOID:363/uterine cancer;DOID:1793/pancreatic cancer;DOID:1993/rectum cancer
Q685J3	MUC17	Mucin-17	2784	T	N	DOID:2394/ovarian cancer;DOID:11934/head and neck cancer;DOID:0060119/pharynx cancer
Q6P1J6	PLB1	Phospholipase B1, membrane-associated	645	D	N	DOID:363/uterine cancer;DOID:0060119/pharynx cancer;DOID:11934/head and neck cancer
Q6UWW8	CES3	Carboxylesterase 3	161	D	N	DOID:4159/skin cancer;DOID:219/colon cancer;DOID:5672/large intestine cancer
Q6UX06	OLFM4	Olfactomedin-4	372	R	S	DOID:1793/pancreatic cancer;DOID:1612/breast cancer;DOID:5041/esophageal cancer
Q7Z304	MAMDC2	MAM domain-containing protein 2	319	D	N	DOID:4159/skin cancer;DOID:1612/breast cancer;DOID:11054/urinary bladder cancer
Q7Z5H5	VN1R4	Vomeronasal type-1 receptor 4	265	L	S	DOID:4362/cervical cancer;DOID:1793/pancreatic cancer;DOID:11934/head and neck cancer
Q86TH1	ADAMTSL2	ADAMTS-like protein 2	44	D	N	DOID:10534/stomach cancer;DOID:219/colon cancer;DOID:5672/large intestine cancer
Q8IZD2	KMT2E	Histone-lysine N-methyltransferase 2E	902	D	N	DOID:363/uterine cancer;DOID:219/colon cancer;DOID:5672/large intestine cancer
Q8N158	GPC2	Glypican-2	200	D	N	DOID:1612 / breast cancer;DOID:5041/esophageal cancer;DOID:5672/large intestine cancer
Q8N8F6	YIPF7	Protein YIPF7	141	D	N	DOID:4362/cervical cancer;DOID:0060119/pharynx cancer;DOID:11934/head and neck cancer
Q8NFZ4	NLGN2	Neuroligin-2	542	A	T	DOID:8618/oral cavity cancer;DOID:0060119/pharynx cancer;DOID:11934/head and neck cancer
Q8NGZ4	OR2G3	Olfactory receptor 2G3	159	H	N	DOID:363/uterine cancer;DOID:219/colon cancer;DOID:1324/lung cancer
Q8TC71	SPATA18	Mitochondria-eating protein	404	K	N	DOID:363/uterine cancer;DOID:1319/brain cancer;DOID:1793/pancreatic cancer;DOID:1324/lung cancer
Q8TDM6	DLG5	Disks large homolog 5	1799	D	N	DOID:363/uterine cancer;DOID:0060119/pharynx cancer;DOID:11934/head and neck cancer
Q92556	ELMO1	Engulfment and cell motility protein 1	55	D	N	DOID:4159/skin cancer;DOID:1319/brain cancer;DOID:3070/malignant glioma
Q92736	RYR2	Ryanodine receptor 2	898	I	T	DOID:0060119/pharynx cancer;DOID:1324/lung cancer;DOID:11934/head and neck cancer
Q96PZ7	CSMD1	CUB and sushi domain-containing protein 1	3053	D	N	DOID:4159/skin cancer;DOID:10534/stomach cancer;DOID:219/colon cancer
Q9BXX0	EMILIN2	EMILIN-2	759	K	N	DOID:363/uterine cancer;DOID:1324/lung cancer;DOID:11054/urinary bladder cancer
Q9H2B2	SYT4	Synaptotagmin-4	89	K	N	DOID:363/uterine cancer;DOID:1612/breast cancer;DOID:219/colon cancer
Q9H9P2	CHODL	Chondrolectin	186	P	S	DOID:4159/skin cancer;DOID:1612/breast cancer;DOID:1324/lung cancer
Q9UKJ8	ADAM21	Disintegrin and metalloproteinase domain-containing protein 21	278	D	N	DOID:4159/skin cancer;DOID:219/colon cancer;DOID:5672/large intestine cancer
Q9UQP3	TNN	Tenascin-N	1091	D	N	DOID:0060119/pharynx cancer;DOID:1324/lung cancer;DOID:11934/head and neck cancer
Q9Y5F1	PCDHB12	Protocadherin beta-12	556	D	N	DOID:363/uterine cancer;DOID:4159/skin cancer;DOID:10534/stomach cancer

NOTCH1 is a member of the highly conserved Notch signaling pathway, responsible for cell fate during development and homeostasis^70,71^. Extensive N- and O-linked glycosylation has been observed in the Notch extracellular domain^71^, where ligand binding induces conformational change and functional receptor activation^72^. A variety of roles have been described for Notch with respect to carcinogenesis and cancer stem cells^73^. We observed the NOTCH1 A465T variant in colon cancer [DOID:219], malignant glioma [DOID:3070], esophageal cancer [DOID:5041], head and neck cancer [DOID:11934], pharynx cancer [DOID:0060119], and brain cancer [DOID:1319].

Mucin-2 is known to be densely O-glycosylated^74,75^, and has been observed with downregulated expression in colorectal cancer^76^. In fact, expression of MUC2 has been associated with less aggressive forms of urothelial bladder cancer^28^. While there are many studies regarding MUC2 expression in cancer^77^ and its variable O-glycosylation patterns^78^, a direct link to N-glycosylation-altering-variation does not exist in current literature: this fact, combined with a potential gain of N-glycosylation variant in seven cancer types, warrants additional mechanistic exploration for the role of MUC2 in cancer. We observed the MUC2 T1750N variant in breast cancer [DOID:1612], liver cancer [DOID:3571], thyroid cancer [DOID:1781], ovarian cancer [DOID:2394], prostate cancer [DOID:10283], brain cancer [DOID:1319], and rectum cancer [DOID:1993].

#### Significant sequons

The protein with the highest number of somatic-only LOGs is Usherin (USH2A, O75445). 5,202 amino acids in length, Usherin has 27 LOG variations abolishing 25 high-confidence sequons. Similarly, the protein with the highest number of somatic-only GOGs is Titin (TTN, Q8WZ42) with 92 GOG predicted variations affecting 90 positions. However, this protein is annotated to be localized mostly to the cytoplasm or nucleus and does not have a signal peptide, implying that glycosylation is unlikely. Low-density lipoprotein receptor-related protein 1B (LRP1B, Q9NZR2) is a membrane protein with a signal peptide and has been observed to have recurrent mutations in urachal cancer^79^ and ovarian clear cell carcinoma^80^. LRP1B is commonly deleted across cancers^81^ and it has been proposed to be a tumor suppressor via modulation of the extracellular tumor environment in thyroid cancer cells^82^. In our dataset, LRP1B has 24 predicted somatic-only GOGs affecting 23 positions. Although no literature exists regarding glycosylation of LRP1B in cancer, its known participation in multiple cancers suggests that altered glycosylation of the protein could affect the course of the disease.

#### Significant proteins

If we divide the number of affected sequons per protein by the length of the corresponding protein, we can identify the proteins with the highest density of nsSNV-affected NLGs per unit length. Considering only the proteins that are annotated with keywords “secreted” or “membrane,” in the LOG set, the top-ranked protein by density is Carcinoembryonic antigen-related cell adhesion molecule 7 (CEACAM7, Q14002) with five mutations abolishing sequons at five distinct positions. CEACAM7 belongs to the immunoglobulin superfamily, specifically to the carcinoembryonic antigen (CEA) gene family, and normally functions in regulation of cellular differentiation^83^. UniProtKB reports CEACAM7 to be strongly downregulated in colonic adenocarcinomas, and it has been reported to be a predictive marker for rectal cancer recurrence^84^. Among the five somatic-only variations leading to LOG on CEACAM7 three are observed in skin cancer, the other two in prostate and pharynx cancers, independently. Although this protein only has five LOG variants, it is a relatively small protein (265 residues in length) resulting in a density of one LOG per every 53 residues. Another CEA family gene, Carcinoembryonic antigen-related cell adhesion molecule 5 (CEACAM 5, P06731), also has one of the highest densities of somatic-only LOG variants. CEACAM5 has 12 variants occurring at 11 positions, for a density of one LOG per every 64 residues. Six of the variants are observed in skin cancer, with others reported in colon, kidney, uterine, ovarian, and hematologic cancers. In the somatic-only GOG set, the top-ranked protein by density is Cellular tumor antigen p53 (TP53, P04637), with 15 GOG variants for an average of one per every 27 residues. While mutation of TP53 is well-characterized to interrupt its normal transcription factor function and lead to the development of many different cancers^85^, its cytoplasmic and nuclear localizations, as well as lack of signal peptide, make it an unlikely candidate for biologically viable N-glycosylation. Epididymal-specific lipocalin-8 (LCN8, Q6JVE9) is a secreted protein with a signal peptide with a density of one GOG variant for every 59 residues. Its three GOG variants are reported in liver and skin cancer datasets, but there is no current literature linking this gene to cancer.

### Pathway and biomarker analysis summary

#### LOG/GOG-cancer associations

Of 8,894 distinct LOG somatic-only variants, only 20 were indicated in publications reported from HGMD (Human Gene Mutation Database, June 2016 release)^86^, only one of which is directly cancer-related (CREBBP Q92793 N1978D; ovarian cancer) and another nine with cancer-related syndromes. Similarly, of 12,939 distinct GOG somatic-only variants, only 16 overlap with HGMD disease annotations, none of which are directly cancer-related, and two of which confer increased risk of certain cancers. With respect to germline variants mapping to the LOG and GOG subsets, we see 349 LOG-associated germline variants and 436 GOG-associated germline variants mapping to HGMD mutations, out of a total of 40,365 HGMD mutations found in the entire nonsynonymous germline dataset retrieved from dbSNP. We hypothesize that mutations already associated with any disease (not restricted to cancer alone) identified in our somatic-only datasets could become high-value biomarkers for different cancer types based on existing literature evidence for functional disruption. At the same time, the minimal overlap between the HGMD dataset and our somatic-only findings suggests enormous potential for validation of as yet unpublished cancer-associated LOG/GOG-causing variants. (See Supplemental Tables S7 and S8 for more information.)

#### Enrichment analysis

The top five canonical MetaCore™ pathways enriched across the set of all cancer-associated NLG variations (including both LOG- and GOG-causing variants) include muscle contraction GPCRs in the regulation of smooth muscle tone (*P* = 2.120e-09), signal transduction mTORC2 downstream signaling (*P* = 1.058e-8), development regulation of epithelial-to-mesenchymal transition (EMT) (*P* = 1.477e-8), breast cancer (general schema) (*P* = 9.996e-8), and nociception nociceptin receptor signaling (*P* = 1.259e-7). Interestingly, protein folding and maturation POMC processing (*P* = 2.373e-10) is strongly enriched in the GOG dataset, including 19/30 associated pathway genes, but having only two genes in common with the corresponding LOG dataset and no additional genes unique to the LOG dataset. This implies a strong bias for this pathway in potential pathogenesis of cancer associated with GOG mutations. The top five pathways for the GOG somatic variants (considered independently of any potential LOG overlap, ranked by statistical significance) are cytoskeleton remodeling_TGF, WNT and cytoskeletal remodeling (*P* = 4.857e-12), cytoskeleton remodeling_cytoskeleton remodeling (*P* = 4.778e-11), ovarian cancer (main signaling cascades) (*P* = 1.668e-10), cell adhesion_chemokines and adhesion (*P* = 9.465e-10), and development_regulation of cytoskeleton proteins in oligodendrocyte differentiation and myelination (*P* = 1.166e-9). Similarly, the top five LOG somatic variants include signal transduction_mTORC2 downstream signaling (*P* = 4.862e-11), development_regulation of epithelial-to-mesenchymal transition (EMT) (*P* = 1.985e-8), cell adhesion_histamine H1 receptor signaling in the interruption of cell barrier integrity (*P* = 2.192e-8), cell adhesion_cadherin-mediated cell adhesion (*P* = 4.214e-8), and some pathways of EMT in cancer cells (*P* = 1.002e-7).

## Discussion

The primary aims of this study were to: (1) provide a comprehensive view of protein N-linked glycosylation sequons within the human genome; and (2) compare cancer-centric nsSNVs and germline SNPs with respect to their relative impact on protein N-linked glycosylation sequons. Despite the *in silico* nature of this work, the consideration of existing experimental literature and functional annotations as evidence supporting the likelihood of possible glycosylation at various sites will allow downstream users of these findings to prioritize sites for additional study and to provide experimental validation that is currently lacking. With this goal in mind, we aimed to delineate which sites were verified sites of glycosylation and which were not, further classifying un-verified sites as possible and probable based on the evidence available for each site. To this end, we provided a comprehensive survey of human NLGs including up-to-date values for: (1) the number of experimentally verified human N-linked glycosylation sites (16,253, or 27.4% of all NLGs); (2) the proportion of human proteins containing at least one NLG (75.8%, or 15,318 of 20,199 total proteins); and (3) the average number of NLGs per human protein (3.9).

We further aimed to associate possible loss or gain of glycosylation with the development of cancer by looking at the subset of variants expected to cause loss or gain of an NLG in somatic-only cancer variant calls. While loss or creation of an NLG does not necessarily impact N-glycosylation, we used additional functional information (including presence of signal peptides and subcellular locations), existing literature regarding role in N-glycosylation, and structural modeling predictions to devise a high-confidence criterion for likelihood of biologically plausible glycosylation. Even if glycosylation has been observed at a given site, effects on so-called “normal” glycosylation at a single site does not guarantee a functional consequence. However, combining information regarding the pervasiveness of independent variants across multiple cancers with evidence supporting plausible glycosylation allows us to rank variants. We have provided lists of variants (both LOG and GOG) meeting at least one high-confidence requirement for glycosylation and appearing in at least three cancers (Supplemental Tables S9 and S10). We suggest prioritizing these variants for additional study including possible experimental validation of N-glycosylation. While the change of the glycosylation status itself may not be pathogenic, the complexity of N-glycosylation and its potential impact on a number of factors may warrant multi-faceted exploration to determine whether a possible association with cancer could be directly explained by simple variation, loss or gain of glycosylation, or downstream effects of altered glycosylation. We expect that these cancer-associated, high-confidence, LOG/GOG-causing variants may lead to biomarker development and help to better elucidate the role of glycosylation in carcinogenesis.

## Materials and Methods

### Data retrieval and integration

Somatic nsSNVs were collected from COSMIC (CosmicCompleteExport_v73), IntOGen (Release-2014-12), ICGC (Data Release v0.10a), TCGA (Release-2015-01-27), ClinVar (ClinVarFullRelease_2015-02-05), and literature mining methods^87^; germline SNPs were collected from dbSNP (Human Build 149). Cancer-related somatic nsSNVs were integrated using the previously described BioMuta workflow^23^ for pan-cancer analysis such that all cancer types were mapped to DO terms by corresponding disease ontology IDs (DOIDs)^62^. Annotated sequence functional site data for N-linked glycosylation retrieved from UniProtKB/Swiss-Prot (release-2015_01), HPRD 9.0^88^, dbPTM 3.0^89^, neXtProt release 2017-01-21^90^ and NCBI-CDD (2015_Jan)^91^ were treated as high-confidence results. For the purpose of this paper, high-confidence NLGs are those which are annotated as a glycosylation site in a UniProt FT line associated with a PMID or manual assertion, or reported directly from the other databases listed above.

### Identification of real and predicted human NLGs

The identification of all possible human NX(S/T) (X! = P) sequons was performed by three methods. The reference UniProtKB/Swiss-Prot human proteome was obtained in January 2015 from the UniProt ftp site at ftp://ftp.uniprot.org/pub/databases/uniprot/previous_releases/release-2015_01/knowledgebase/knowledgebase2015_01.tar.gz, and annotated N-linked glycosylation sites were retrieved from this set of protein entries. Additionally, the NetNGlyc-1.0 [http://www.cbs.dtu.dk/services/NetNGlyc/] tool was used to predict possible N-linked glycosylation sites. Finally, all NX(S/T) (X! = P) sequons in the human proteome were identified by searching all human protein sequences using custom Python scripting.

### Calculating frequency of human NLG occurrence and occupancy rate

The proportion of NLG-containing human proteins was reported as the number of human proteins in UniProtKB/Swiss-Prot with at least one NLG divided by the total number of human proteins in UniProtKB/Swiss-Prot. Frequency of NLGs was calculated as the total number of human NLGs divided by the total number of human proteins in UniProtKB/Swiss-Prot. Occupancy was calculated as the number of real N-linked glycosylation sites divided by the number of human NLGs in UniProtKB/Swiss-Prot.

### Mapping NLGs to variations and reporting of LOG/GOG

**Figure 5:**
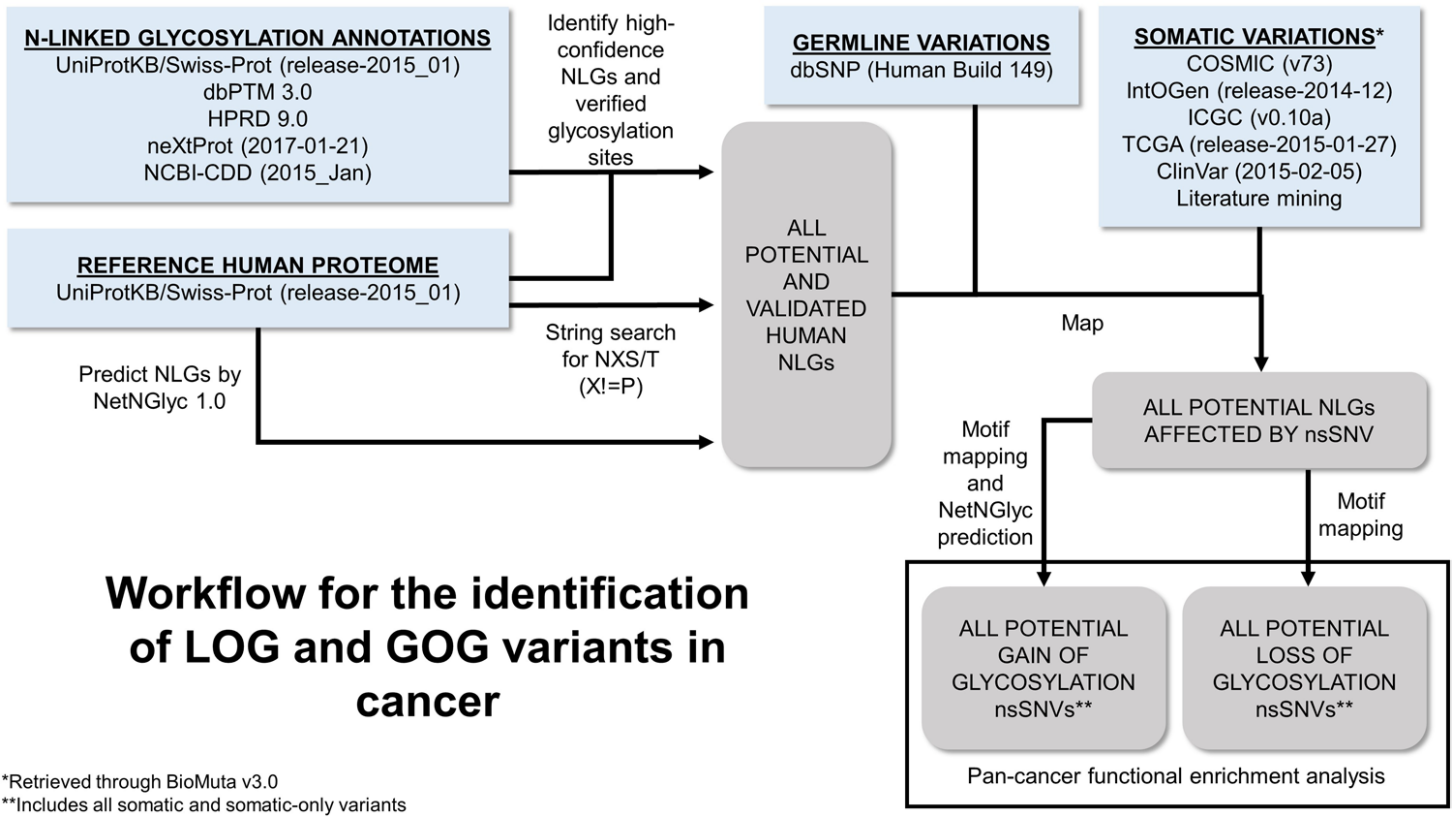
Flowchart of the identification of LOG and GOG. The complete human proteome was retrieved from UniProtKB/Swiss-Prot, and sequences of included proteins were analyzed by string search and by NetNGlyc to identify all potential NLGs. High-confidence annotations of NLGs were also retrieved from the specified databases and incorporated into the comprehensive NLG dataset. NLGs were then mapped to somatic nsSNVs reported by cancer genomics databases and germline variations reported by dbSNP. The impact of variation on NLGs was analyzed, and for the subset resulting in loss or gain of NLG (LOG and GOG, respectively), presence in cancer samples was reported.

Each potential NLG was mapped to variation datasets and reported as LOG or GOG. An altered NLG was considered a LOG if the altered version had an N abolished at the first position, an S or T abolished at the third position, or a newly generated P at the second position. Following the rationale of our previous study^55^, variants that alter T to S (or S to T) at the third position were not considered to functionally affect NLGs. An altered NLG was considered a GOG if the altered version contained a newly generated N at the first position, a newly generated S or T at the third position, or an abolished P within a normal NPS/T subsequence. Variants were also mapped to the human proteome and analyzed using NetNGlyc to predict additional GOGs. Figure 5 shows the workflow for the LOG/GOG identification and variation mapping processes. It should be noted that the NetNGlyc tool may predict a GOG by modification of a residue outside the sequon and may also predict GOG including P at the middle sequon position: the number of GOGs suggested as candidates for further analysis was based on overlap between simple mapping results and NetNGlyc predictions.

### LOG and GOG identification and pan-cancer analysis

Significant variations, sequons, and proteins were identified according to the mapping results for LOG and GOG, especially for variations observed in multiple types of cancer. To assess the potential enrichment of a given feature and discrepancy of occurrence of that feature between datasets, a binomial test was used as described in an earlier study^92^. For example, to determine the *P-value* for significance of abolished sequons, the total number of abolished sequons across all methods was used to determine the expected rate of abolished sequons; this rate was then compared to the observed rate of abolished sequons for a given method. To compare somatic and germline variations, we used the somatic-only nsSNVs and germline SNPs and excluded any overlapping variations between the two datasets. Numbers of associated cancer types were counted by summing distinct DOIDs annotated at the variant level. Note that DOID mapping in BioMuta was done only for the subset of cancer-related DOIDs (CDO slim)^62^. Since some samples are of a particular cellular subtype, they may automatically map to multiple terms designating tissue-level and cell-level specificity separately.

### HGMD comparison

Our set of cancer-related, NLG-impacting variants was compared to variants in the Human Gene Mutation Database (HGMD, HGMD_Professional_2016.2)^93^. Because HGMD includes only published gene lesions, any identified nsSNV-affected LOG/GOG not cross-referenced by HGMD represented possible novel findings.

### Enrichment analysis

Enrichment of pathways was analyzed with MetaCore™ (https://portal.genego.com).

### Data availability

All data generated or analyzed during this study are available as supplemental tables accompanying this publication and can be browsed and downloaded from https://hive.biochemistry.gwu.edu/kbdata/view/loss_and_gain_of_n_linked_glycosylation_sequons_in_cancer by selecting tables with prefix “NLGPaper” from the dropdown menu.

## Electronic supplementary material

Table S1

Table S2

Table S3

Table S4

Table S5

Table S6

Table S7

Table S8

Table S9

Table S10
